# Ultra short time to Echo (UTE) MRI for cephalometric analysis–Potential of an x-ray free fast cephalometric projection technique

**DOI:** 10.1371/journal.pone.0257224

**Published:** 2021-09-13

**Authors:** Ciamak Abkai, Jan Hourfar, Jörg Glockengießer, Johannes Ulrici, Erich Hell, Volker Rasche, Björn Ludwig

**Affiliations:** 1 Dental office, Traben-Trarbach, Germany; 2 Sirona Dental Systems GmbH, Bensheim, Germany; 3 Department of Orthodontics, Saarland University, Homburg/Saar, Germany; 4 Dental office, Salzburg, Austria; 5 Department of Internal Medicine II, Ulm-University, Ulm, Germany; McLean Hospital, UNITED STATES

## Abstract

**Objectives:**

A novel magnetic resonance imaging (MRI) scan protocol is presented on the basis of ultra-short time to echo (UTE). By this MRI cephalometric projections (MCPs) can be acquired without the need of post processing in one shot. Different technical parameterizations of the protocol are performed. Their impact on the performance of MCPs is evaluated in comparison to the gold standard–the lateral cephalometric radiography (LCR) for cephalometric analysis (CA) in orthodontics.

**Methods:**

Seven MCPs with various scan parameters influencing the scan duration and one LCR are used from one subject. 40 expert assessors performed CA for 14 predefined cephalometric landmarks. Relative metric distances and absolute angular measurements were calculated. Statistical analysis is presented and the deviations are highlighted to demonstrate the potential of the method for further analysis.

**Results:**

The MCPs are acquired in 5–154 seconds, depending on resolution and contrast. Mean relative distances were 2.4–2.7 mm in MCPs and 1.6 mm in LCR, which demonstrate the accuracy and level of agreement of the expert assessors in identifying anatomical landmarks. In comparison to other studies, the presented MCP performed similar in angular analysis and demonstrated on average deviation of 1.2° ±1.1° in comparison to LCR. Despite the point articulare (Ar) and the related gonial angle the calculate distances and angles show outcomes in the range of ±2°/2mm.

**Conclusions:**

MCPs can be acquired much faster in comparison to other techniques known from literature for CA. This study demonstrated the potential of the new method and showed first feasible results. Further research is needed to analyze the performance on a broad range of patients.

## Introduction

Lateral cephalometric radiography (LCR) also known as cephalograms (CEPH) are used as a standard tool in orthodontics. Preferably using angular measurements, cephalometric analysis (CA) can be performed on the LCR [1] and is used for the assessment of treatment planning, evaluation and follow-up [2, 3]. Nowadays, more than 100 different CAs are available using a large diversity of mostly bony anatomical landmarks [4], representing either median or paramedian anatomical structures on the skull.

X-ray techniques such as LCRs, either conventional or digital, are inherently associated with radiation burden. A typical LCR exposes the patient with up to 5–6 micro Sievert (μSv) [5]. Because most orthodontic patients are adolescents in active growth, who are particularly susceptible to the effects of radiation [6], magnetic resonance imaging (MRI) could be a radiation-free alternative which is getting increasingly important and available. In orthodontics, MRI has been well used in studies to investigate treatment related alterations of the temporo-mandibular joint (TMJ) [7].

In 2012, Eley et al. introduced a gradient echo (GRE) scan protocol with low flip-angle and reduced repetition time (TR) and echo time (TE), known as “black-bone”, which led to a considerable bone soft-tissue contrast in volume MRI images [8]. In 2013, Eley et al. used the same protocol for the acquisition of two-dimensional (2D) midsagittal planes and compared them traditional T1- and T2- weighted images as well as to LCR, which they defined as gold-standard [9]. Although the image’s field of view was reduced from three-dimensional (3D) to a single slice (2D), anatomical information from para-median planes was not available.

In 2017, Heil et al. overcame this issue by cropping manually selected image information from eight areas including para-median planes into one final image [10]. They conducted a T1-weighted isotropic 3D volume acquisition using parallel imaging technique with an acquisition time of 6:59 minutes. The protocol was based on a Turbo Spin Echo (TSE) technique, which is also know from MRI based orthodontic diagnosis [11].

In this study a novel scan protocol is introduced, which provides MRI cephalometric projections (MCP) in one shot, including information from median and para-median information in terms of a real orthogonal projection without the need of post-processing or cropping. The protocol further reduces repetition and echo times towards an Ultra Short Echo-time (UTE) modality, which provides a high bone soft-tissue contrast in comparison to other acquisition techniques [12]. By this the acquisition time was significantly reduced, while sagittal resolution was considerably higher than in previous protocols. A large panel of experienced assessors was asked to identify anatomical landmarks relevant for CA in order to demonstrate the first feasibility of this approach.

## Materials and methods

### Ethical approval

Ethical approval for this study was obtained (IRB No.162-12, University of Ulm, Ulm, Germany).

### Imaging

A fully dentate adult male patient without contra-indication for MRI consented to participate in our preliminary investigation. The patient’s skull was free of metal such as dental implants or osteosynthesis material. Some teeth exhibited very small metal restorations. The x-ray derived digital LCR (Orthophos® SL, Sirona, Bensheim, Germany) was available as part of mandatory initial records prior to orthodontic treatment. Device settings during exposure were 77 kV, 14 mA and 23 mGycm^2^. Scan time was 9.2 seconds. Size of the LCR was 1804 x 2136 pixels; pixel size was 0.095 mm x 0.095 mm measured at the calibration ruler projected onto the LCR. 7 MCPs were acquired from the same patient using a 3 tesla system (Philips Achieva, Philips Healthcare, Hamburg, Germany) with an eight channel head coil. The MCP fast projection protocol allowed for comparably short scan durations ranging from 5 to 152 seconds depending on sequence specific scan parameters (**Table 1**).

**Table 1:** MCP acquisition details for images i.

i	Pixel size	Field of view	Scan time	TE	TR	Pixel bandwidth	NEX
	[mm x mm]	[mm x mm]	[s]	[μs]	[ms]	[Hz]	
1	0.39x0.39	293.3 x 293.3	5	358	4.2	816	1
2	0.39x0.39	293.3 x 293.3	16	412	6.3	517	1
3	0.39x0.39	293.3 x 293.3	30	382	6.3	517	1
4	0.39x0.39	293.3 x 293.3	59	389	7.2	517	1
5	0.2 x 0.2	300 x 300	73	361	15.2	259	1
6	0.39x0.39	293.3 x 293.3	123	373	8.0	517	2
7	0.2 x 0.2	300 x 300	154	360	16.9	259	1

TE = Time to Echo, TR = Repetition time, NEX = Number of excitations/averages.

The flip angle was adjusted to 5° for all MRI images to guarantee a fast spin relaxation in order to achieve short scan times. Thick slice selection (80 mm) was realized by exiting more area in one step in a radial scheme coding. Slice orientation was adjusted manually on the basis of a navigator scout and was set to orthogonal to the sagittal plane. Geometric accuracy was confirmed for the scanner on the basis of phantom measurements. Assuming linearity and homogeneity of the gradient coil encoding fields, the resulting MCP represents an orthogonal projection to the slice encoding plane. Image post processing was neither necessary nor applied on the MCPs.

### Expert assessors and landmark identification

The set of 8 images (1 LCR and 7 MCP) was presented to a panel of 40 orthodontists using a web server. All of them had at least 15 years of experience in CA. The first seven images to assess were the MCPs, which have been arranged in random order; the last image of the series was always the LCR. To avoid any preconditioning, the assessors had not been introduced or trained in MRI and especially in our protocol. The assessors were asked to identify 14 commonly used cephalometric landmarks (**Table 2**) on each of the 8 images, utilizing a customized software tool running on the web server. To ensure optimal tracing conditions for each individual assessor, image enhancement features zoom in/out, change of brightness and contrast were provided. For all images, all landmarks were recorded with two digit accuracy as x- and y-coordinates (in millimeters) in relation to the top left corner as reference.

**Table 2:** Landmark description.

ID	Landmark	Definition
N	Nasion	The most anterior point in the frontonasal suture in the mid-sagittal plane
S	Sella	The geometric centre of the pituitary fossa
Ar	Articulare	Intersection of the inferior surface of the cranial base and the posterior outlines of the ascending rami or mandibular condyles
TPPBR	Posterior Tangent Point	Tangent point of the posterior border of the ramus
TPIBR	Inferior Tangent Point	Tangent point of the inferior border of the ramus
Me	Menton	The lowest point on the symphyseal shadow of the mandible seen on a lateral cephalogram
Pg	Pogonion	The most anterior point in the contour of the chin in the sagittal plane.
-1apex	-1apex	Apex of the lower central incisor
-1li	Incisioninferius	The point of the lower incisor farthest from the apex of the root.
+1apex	+1apex	Apex of the upper central incisor
+1ls	Incisionsuperius	The point of the lower incisor farthest from the apex of the root.
A	A-point	The most posterior midline point in the concavity between ANS and the most inferior point on the alveolar bone overlying the maxillary incisors
ANS	Anterior nasal spine	The anterior tip of the sharp bony process of the maxilla at the lower margin of the anterior nasal opening
PNS	Posterior nasal spine	The posterior spine of the palatine bone constituting the hard palate

### Data collection

The data collection was undertaken as following:

- Images *i*∈{1,..,*I*}, *I* = 8, whereas the image with ID *i* = 8 is the LCR image, which has been considered as the reference image. The images *i* = 1–7 represent the MCP images with ascending scan time (compare to **Table 1**).
- Assessments *a*∈{1,…,*A*} *A* = 40.
- Points *p*∈{1,..,*P*} *P* = 14 representing the landmark points (compare to **Table 2**), while each point *p* is recorded as a pair of coordinates *p* = (*x*_*p*_, *y*_*p*_) with dimension [mm, mm].

As a result, 4480 pairs (*x*,*y*)(*i*,*a*,*p*)∈*IxAxP* of data have been collected in this study for further analysis.

### Metric evaluation

The mean landmark position ${\left(\bar{x},\bar{y}\right)}_{i,p}$ (**Eq 1**) was calculated for each landmark point *p* in the respective image *i* on the basis of all coordinates (*x*,*y*) selected by assessors *a*∈{1,…,*A*} *A* = 40.


$${\left(\bar{x},\bar{y}\right)}_{i,p}=1/R(\sum_{a=1}^{A}x(i,a,p),\sum_{a=1}^{A}y(i,a,p))$$ (Eq 1)

On this basis the relative Euclidian distance *d*(*a*) (in mm) for each point *p* in an image *i* was defined according to **Eq** 2.


$$d{\left(a\right)}_{i,p}=\sqrt{{\left(x(i,a,p)-\bar{x}\right)}^{2}+{\left(y(i,a,p)-\bar{y}\right)}^{2}}$$ (Eq 2)

### Angular evaluation

A selection of 10 commonly used cephalometric angles (**Table 3**) were calculated for each image. The angle (*α*) between two lines, defined by 4 corresponding landmark positions *p*_1_..*p*_4_, was calculated according to **Eq** 3.


$$\alpha (\vec{p_{1}p_{2}},\vec{p_{3}p_{4}})=\mathrm{arcos}\left(\frac{\vec{p_{1}p_{2}}∙\vec{p_{3}p_{4}}}{\left|\vec{p_{1}p_{2}}|∙|\vec{p_{3}p_{4}}\right|}\right)*\frac{180}{\pi }$$ (Eq 3)

**Table 3:** Description of the angular measurements of the cephalometric analysis.

Notation	Technical description according to Eq 2	Description
S-N-A	$\alpha (\vec{SN},\vec{NA})$	Maxillary position
S-N-Pg	$\alpha (\vec{SN},\vec{N\;Pg})$	Mandibular position
A-N-Pg	$\alpha (\vec{AN},\vec{N\;Pg})$	Sagittal jaw relation
S-N/ANS-PNS	$\alpha (\vec{SN},\vec{ANS\;PNS})$	Maxillary inclination
S-N/ TPIBR-Me	$\alpha (\vec{SN},\vec{TPIBR\;Me})$	Mandibular inclination
ANS-PNS/TPIBR-Me	$\alpha (\vec{ANS\;PNS},\vec{TPIBR\;Me})$	Vertical jaw relation
Ar-TPPBR/TPIBR-Me	$\alpha (\vec{Ar\;TPPBR},\vec{TPIBR\;Me})$	Gonial angle
+1/ANS-PNS	$\alpha (\vec{+1ls+1apex},\vec{ANS\;PNS})$	Maxillary incisor inclination
-1/ TPIBR-Me	$\alpha (\vec{-1li-1apex},\vec{TPIBR\;Me})$	Mandibular incisor inclination
+1 /-1	$\alpha (\vec{+1ls+1apex},\vec{-1li-1apex})$	Interincisal angle

By this, differences between the reference LCR („gold standard“) and MCPs could be evaluated on the basis of absolute and relative measurements.

### Statistical analysis

Normal distribution of the data was confirmed using the Kolmogorov-Smirnov-Test. Homogeneity of variance was evaluated with Levene’s method. Parametric testing was undertaken by two-tailed t-tests with significance level *α* = 0.05. Descriptive mean (M) and standard deviation (SD) were calculated for the different variables. Null hypothesis was that the mean values were equivalent. The MCP and LCR have been considered as equivalent, if the null hypothesis was fulfilled and statistical difference was not significant (p-value>0.05). Statistical analyses were carried out with SPSS® for Windows, version 22.0 (IBM Corp., Armonk, New York, USA).

## Results

### MCPs

**Fig 1:**
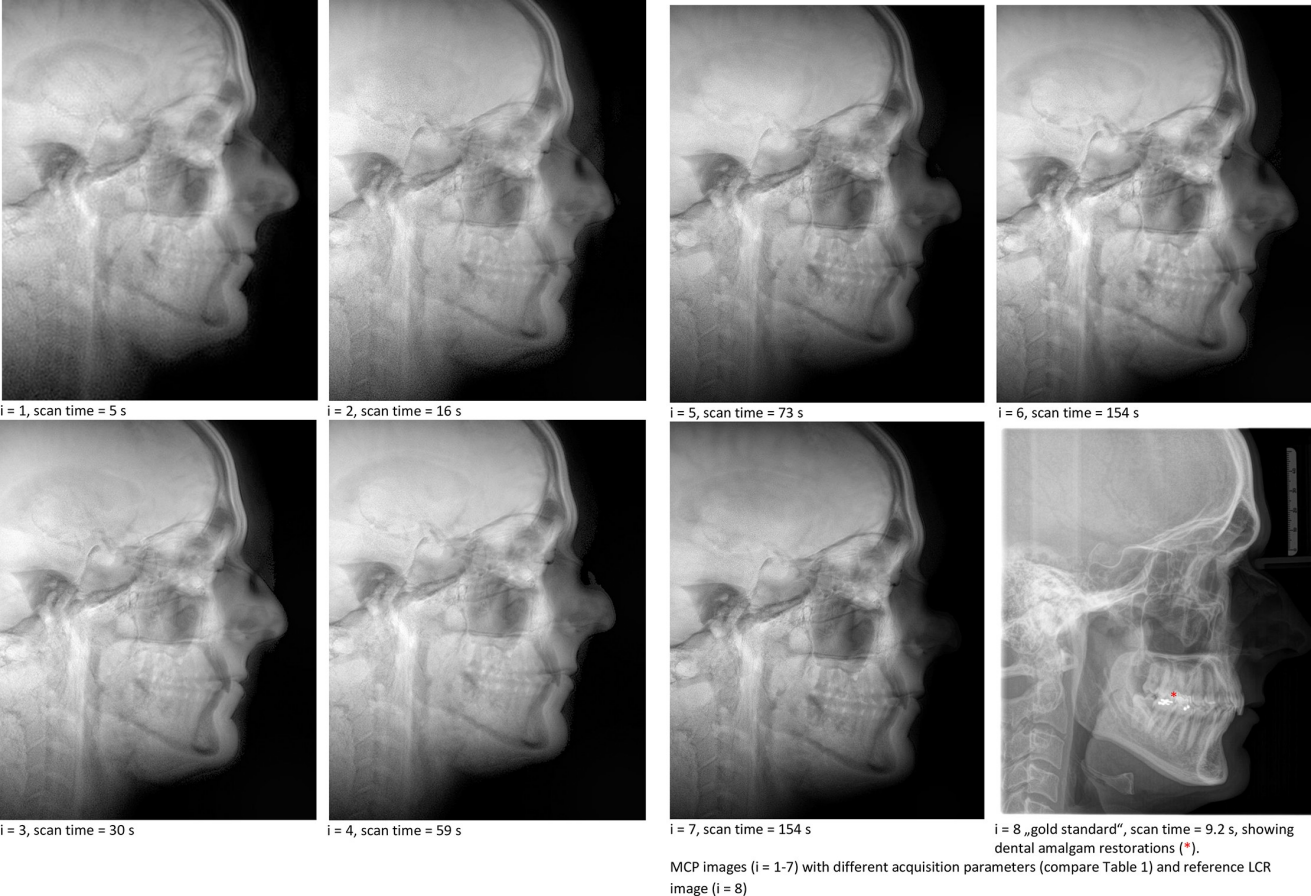
MCP images (i = 1–7) with different acquisition parameters (compare Table 1) and reference LCR image (i = 8).

MCPs (i = 1..7) are depicted in comparison to LCR (i = 8) in **Fig 1**.

### Metric measures

The statistical evaluation of the relative metric measurements is shown in detail in **Table 4**, according to **Eq 2**. For the MCPs, the relative distance *d*(*a*) varied considerably for the different cephalometric landmarks in images i = 1..7 on average (a = 1..40) with mean values in the range 0.5–5.9 mm in contrast to 0.4–2.2 mm in LCR (without considering *TPPBR*, *TPIBR*). *TPPBR* and *TPIBR* showed large distance (2.8–5.1 mm for MCP and 2.5/4.3 in LCR) values in both modalities, since these are tangential construction points. Especially landmarks *Ar*, *ANS*, *PNS* and *N* showed higher distance values in MCPs. For these points the difference was statistical significant (p-value in the range 0–0.41) (). The difference was not statistical significant for *TPPBR*, *TPIBR*, *Me*, *Pg*, *+1apex* and *A* for all MCP images (p-value in the range 0.05–0.96) and partially for other landmarks. The average relative distance for all landmarks (i.e. averaging the different evaluated cephalometric landmarks for each image) was very similar for all MCPs (in the range (2.4–2.7)±(2.5–2.9)mm), but was greater than for LCR (1.6±2.0 mm). An additional spread of approximately 1 mm was observable in this study on average between MCPs and LCR. No major trend of metric improvements in MCPs from i = 1–7 in terms of statistical significance, M or SD was noted on average. Particularly, image i = 2 showed the smallest M and SD averaged over all landmarks (2.4±2.5) in comparison to 2.5–2.7±2.7–2.9 mm for the other MCPs and 1.6±2.0 for LCR.

**Table 4:** Relative metric measurements. Mean (M) and standard deviation (SD) of relative distance values in mm (Eq 2) and corresponding p-values for each landmark population. Statistical equivalence was assumed for p-value>0.05 (gray highlighted).

		MCPs																					LCR	
	Image i =	1			2			3			4			5			6			7			8	
		M	SD	p-value	M	SD	p-value	M	SD	p-value	M	SD	p-value	M	SD	p-value	M	SD	p-value	M	SD	p-value	M	SD
Landmark	N	2.8	1.2	0.000	2.8	1.6	0.000	3.0	1.2	0.000	3.3	1.5	0.000	3.5	1.5	0.000	3.4	1.5	0.000	3.5	1.7	0.000	1.1	0.9
	S	1.7	2.5	0.021	1.8	1.7	0.000	1.2	1.5	0.049	0.7	0.4	0.761	0.9	0.7	0.077	0.8	0.4	0.139	1.0	1.4	0.124	0.7	0.4
	Ar	5.6	4.4	0.000	3.9	4.2	0.000	5.2	4.6	0.000	5.8	4.7	0.000	5.1	4.5	0.000	5.3	4.6	0.000	5.9	5.1	0.000	1.0	0.6
	TPPBR	4.9	3.9	0.567	4.8	4.2	0.648	4.8	4.3	0.669	4.6	4.5	0.786	5.1	5.1	0.501	4.3	4.2	0.960	4.9	4.6	0.587	4.3	4.8
	TPIBR	3.8	3.7	0.095	3.1	2.8	0.356	3.2	3.7	0.351	3.0	3.8	0.475	3.0	2.8	0.470	3.5	3.7	0.186	2.8	3.5	0.689	2.5	3.3
	Me	1.7	1.7	0.398	1.9	2.1	0.416	2.0	2.1	0.346	2.2	2.3	0.189	2.0	1.8	0.322	2.1	2.3	0.231	2.0	1.9	0.311	1.8	1.9
	Pg	2.2	2.5	0.054	1.6	1.1	0.248	1.9	1.3	0.027	1.5	1.2	0.386	1.8	1.8	0.145	1.6	1.3	0.182	1.9	1.7	0.071	1.3	1.2
	-1apex	1.7	1.1	0.013	2.1	1.5	0.001	2.1	1.1	0.000	2.2	1.1	0.000	2.0	1.7	0.007	2.2	1.3	0.000	2.0	1.1	0.000	1.2	0.7
	-1ie	1.1	0.8	0.000	1.0	0.6	0.000	1.0	0.9	0.003	1.1	0.7	0.000	1.1	0.7	0.000	1.1	0.7	0.000	1.0	0.7	0.000	0.5	0.3
	+1apex	1.8	1.3	0.174	2.4	1.7	0.457	2.7	1.4	0.067	2.4	1.7	0.416	2.5	1.5	0.339	2.5	1.6	0.331	2.6	1.9	0.262	2.2	1.1
	+1ls	0.7	0.3	0.000	0.6	0.3	0.063	0.5	0.2	0.204	0.6	0.4	0.009	0.6	0.4	0.010	0.6	0.6	0.087	0.6	0.5	0.045	0.4	0.2
	A	1.9	1.2	0.582	2.3	1.2	0.191	2.0	1.2	0.837	2.1	1.1	0.642	2.5	1.5	0.081	2.2	1.2	0.410	2.4	1.4	0.125	2.0	0.8
	ANS	3.5	2.2	0.001	3.8	1.7	0.000	4.0	2.1	0.000	3.9	2.2	0.000	4.3	2.2	0.000	4.2	2.1	0.000	4.2	2.2	0.000	2.1	1.1
	PNS	2.7	1.7	0.000	2.0	2.3	0.041	1.7	1.9	0.112	1.8	2.1	0.062	2.1	2.4	0.023	2.5	2.4	0.002	3.5	2.6	0.000	1.1	0.8
All	M	2.6			2.4			2.5			2.5			2.6			2.6			2.7			1.6	
	SD	2.7			2.5			2.7			2.8			2.8			2.7			2.9			2.0	

## Cephalometric angles

The statistical evaluation of the cephalometric angles as defined in **Table 3** on the basis of **Eq 3** is shown in detail in **Table 5**. Cephalometric angles showed differences of mean values in the range of -4.5–5.7°. On average the MCP showed a deviation of 1.2°. Larger differences were observable for the *gonial (-5*.*7°*, *p-value<0*.*0001)* and *interincisial* (-4.5°, p-values<0.0001) angles. Although the SD for these angles in the LCR was comparable high (2.6–2.8°) the t-test was not seen as non-significant (p-valu>0.05) for any of the MCPs for these angles. On the other hand statistical equivalence was given for *S-N-Pg* and in all MCPs and in some MCP for other angular values. Especially MCPs with higher resolutions (i = 5 and i = 7) showed more angles with non-significant statistical difference to LCR. The mean deviations of cephalometric angles of the MCP (i = 1..7) to LCR (i = 8) ranged between 1.6° ± 1.4° for the 5 second scan (i = 1) and 0.9° ± 1.2°for the 154 second scan (i = 7).

**Table 5:** Analysis of angular measurements. Δ: difference to LCR/gold standard image i = 8; MAD: Mean absolute difference to gold standard (image i = 8); M: mean; SD: standard deviation; p-values >0.05 of t-test were considered as non significant statistical deviation (gray highlighted).

Image i =	1			2			3			4			5			6			7			1–7	8
Angular measurements [°]	M (SD)	Δ M (SD)	p-value	M (SD)	Δ M (SD)	p-value	M (SD)	Δ M (SD)	p-value	M (SD)	Δ M (SD)	p-value	M (SD)	Δ M (SD)	p-value	M (SD)	Δ M (SD)	p-value	M (SD)	Δ M (SD)	p-value	MAD M (SD)	M (SD)
S-N-A	81.3 (3.9)	-1.3 (2.1)	0.071	80.1 (3.7)	-2.6 (1.9)	0.001	81.5 (3.2)	-0.9 (1.4)	0.084	80.7 (3.3)	-1.8 (1.5)	0.003	81.1 (4.4)	-1.4 (2.6)	0.081	80.7 (3.8)	-1.9 (2.0)	0.007	81.8 (3.6)	-0.7 (1.8)	0.266	1.5 (1.9)	82.5 (1.8)
S-N-Pg	82.4 (2.4)	-0.4 (1.4)	0.343	82.4 (2.4)	-0.4 (1.4)	0.406	83.1 (1.9)	0.5 (0.9)	0.244	82.5 (2.1)	-0.2 (1.1)	0.457	83.0 (2.3)	0.3 (1.3)	0.471	82.6 (2.2)	-0.1 (1.2)	0.736	83.1 (2.1)	0.4 (1.1)	0.275	0.3 (1.2)	82.7 (1.0)
A-N-Pg	1.9 (1.6)	0.8 (0.8)	0.009	2.5 (2.5)	1.4 (1.7)	0.000	2.1 (1.7)	0.9 (0.9)	0.004	2.0 (1.7)	0.9 (0.9)	0.004	2.7 (2.3)	1.6 (1.5)	0.000	2.2 (1.9)	1.1 (1.1)	0.001	2.0 (2.1)	0.9 (1.3)	0.017	1.1 (1.2)	1.2 (0.8)
S-N/	4.2 (2.5)	1.0 (1.2)	0.039	3.4 (2.3)	0.3 (1.0)	0.730	3.1 (2.0)	0.1 (0.7)	0.747	2.9 (2.5)	-0.1 (1.2)	0.561	2.5 (2.0)	-0.6 (0.7)	0.056	2.9 (2.3)	-0.3 (1.0)	0.447	2.7 (1.9)	-0.6 (0.6)	0.207	0.4 (0.9)	3.2 (1.3)
ANS-PNS																							
S-N/	26.8 (2.2)	1.1 (0.1)	0.006	26.6 (3.1)	1.2 (1.0)	0.062	26.7 (2.8)	1.1 (0.7)	0.034	26.3 (2.6)	0.8 (0.5)	0.121	25.9 (2.9)	0.4 (0.8)	0.416	26.2 (2.6)	0.6 (0.5)	0.165	25.9 (2.9)	0.4 (0.8)	0.405	0.8 (0.6)	25.4 (2.1)
TPIBR-Me																							
ANS-PNS/	22.9 (2.6)	0.5 (0.9)	0.335	24.0 (3.1)	1.9 (1.4)	0.009	23.9 (3.4)	1.7 (1.7)	0.015	23.8 (3.5)	1.7 (1.8)	0.023	25.3 (2.9)	3.0 (1.2)	0.000	23.6 (3.3)	1.5 (1.6)	0.043	24.1 (3.0)	1.9 (1.3)	0.003	1.7 (1.4)	22.4 (1.7)
TPIBR-Me																							
Ar-TPPBR/	120.9 (7.6)	-3.4 (7.3)	0.009	123.0 (8.5)	-5.7 (7.8)	0.000	122.3 (7.7)	-4.9 (7.4)	0.000	122.2 (7.8)	-4.9 (7.4)	0.001	120.2 (7.2)	-3.0 (6.3)	0.029	122.0 (6.8)	-4.7 (6.1)	0.000	121.5 (7.5)	-4.1 (7.2)	0.002	4.4 (7.1)	117.3 (2.6)
TPIBR-Me																							
+1/ANS-PNS	107.3 (3.1)	3.5 (1.1)	0.000	105.8 (3.2)	2.2 (1.2)	0.002	105.6 (2.9)	1.8 (0.9)	0.002	104.7 (2.6)	0.9 (0.6)	0.091	103.2 (4.0)	-0.5 (2.0)	0.366	104.7 (2.4)	1.0 (0.4)	0.063	103.8 (3.5)	0.1 (1.5)	0.963	1.4 (1.1)	103.8 (2.0)
-1/ TPIBR-Me	95.1 (2.8)	-2.2 (0.4)	0.000	96.0 (4.0)	-1.6 (1.6)	0.051	98.4 (3.1)	0.8 (0.7)	0.193	97.7 (3.5)	0.2 (1.1)	0.774	97.3 (3.5)	-0.1 (1.1)	0.761	96.8 (4.1)	-0.8 (1.7)	0.316	98.3 (3.4)	0.8 (1.0)	0.256	0.9 (1.1)	97.5 (2.4)
+1/-1	134.7 (3.2)	-1.8 (0.4)	0.008	134.0 (4.5)	-2.5 (1.7)	0.004	132.0 (3.2)	-4.5 (0.4)	0.000	133.7 (3.5)	-2.8 (0.7)	0.000	134.5 (4.1)	-2.1 (1.3)	0.016	134.8 (3.8)	-1.6 (1.0)	0.026	133.8 (3.8)	-2.7 (1.0)	0.001	2.6 (0.9)	136.5 (2.8)
MAD																							Mean SD
over all angles																							i = 8
M	1.4			1.6			1.4			1.0			1.0			1.0			0.9			1.2	
(SD)	(0.9)			(1.4)			(0.9)			(1.0)			(1.4)			(1.2)			(1.2)			(1.1)	1.8

The most accurate angle was *S-N-Pg* with a mean absolute difference of 0.3° ±1.2° and maxillary inclination, which differed by 0.4° ± 0.9°.

## Discussion

### Patient centered acquisition

In young patients the application of the ALARA (as low as reasonably achievable) principle [13, 14] is paramount due to the higher sensitivity of children to radiation. In our study, we employed the UTE protocol as a projection technique by thick slice excitation as a radiation-free imaging modality. This technique leads to first a high contrast between hard and soft tissue and second, provides images in a very fast acquisition mode (in one shot) and significantly reduces the scan time compared to conventional 3D imaging in MRI [9, 10, 18].

Scan times in the range of seconds, depending on protocol parameters, such as resolution or number of averaging (**Table 1**) were comparable to the LCR scan times. In a clinical setting, this may be of great importance. Particularly in young patients with limited compliance, the occurrence of motion artifacts could be reduced [15]. Moreover, our approach produces MCPs without the need for post-processing [10], which may promote integration into clinical routine. Latest developments in this area are very promising to gain a wider field of application also in clinical domain [16–18]. Artifact free orthodontic materials may additionally increase the potential for real clinical routine with benefits in orthodontic treatment planning and control [19].

### MCPs

Various dental and skeletal structures were clearly visible with high contrast in inverted grayscale scheme in comparison to traditional LCR.

Small dental restorations (restorative amalgam) were clearly visible in LCR in contrast to MCPs. No metal induced artifacts were observable in this area, but may be subject to further analysis. Since mainly free protons lead to signal contribution in MRI images, the contrast between areas filled with air (maxillary sinus, trachea, esophagus) and dental or skeletal areas with very small amount of free protons was negligible.

In contrast to typical 3D MRI techniques in our method anisotropic voxels have been used which, represent the orthogonal projection technique. Eley et al. used single slices from a istropic 3D scan and [8] and Heil et al. used manual image fusion of different slices from an isotropic scan [10]. The final outcome in any method will be the reduction of information from the in-plane dimension, which may be acceptable since CA is well-established in 2D.

Increasing resolution (smaller pixel size) and/or higher SNR correspond to longer scan duration and clearly impact the image noise and detail level. Nevertheless, images i = 5 and i = 7 with a pixel size of 0.2 x 0.2 mm^2^ were realized in much faster scan times as reported by similar literature with 3 times higher resolution [10]. By this, the authors report for the first time, MCPs that are realized with a resolution very close to LCR resolution (approx. factor 2 difference) in very short scan times.

This represents an orthogonal projection in comparison to the far-field perspective projection known from LCR.

### Landmark identification and relative metric measures

The traditional LCR on average scored better than the novel MCPs in terms of scattering in landmark identification. Lower resolution, higher noise level, inverted contrast and the lack of training in interpretation of the new type of images are good reasons to explain deviations.

The LCR shows a spread (SD) of 0.2–1.9 mm for all landmarks despite *TPIBR* and *TPPBR*, which demonstrates that the overall agreement of the raters in the known gold standard is very accurate. The *TPIBR* and *TPPBR* are tangential construction points, which are typically not related to point-like anatomical marker. Therefore the spread for the latter landmarks is in all modalities relatively high (SD = 2.8–5.1 mm).

However, certain landmarks were detected with very similar (*+1li*, *-1li)* or smaller (Me, A) mean and standard deviations *comparing MCP* with LCR. The authors expected small scatter for landmark *N*, which should be identified clearly in MCP. However since nasal bone is typically white in LCR in contrast to black in MCP, soft tissue may be a reason of significant higher mean difference for *N*. The largest deviation between LCR and MCP was at point *Ar*. Further improvements of the contrast and investigations in this area are reasonable.

Interestingly, landmark identification did not clearly improve with higher resolution for all landmarks. Only for point *S* better statistical equivalence of mean distances was observed in MCPs with longer scan times. Although the image i = 1 with the shortest scan time (5 s) was the one with the biggest scatter, the resulting relative distance analysis performs very similar to other MCPs. No major differences were observed between image i = 2 (16 s scan time) to i = 7 with the longest scan time (154 s) and the highest resolution on avarage. In fact improving the resolution in MRI will introduce additional noise-level in the image domain, which may be unfamiliar in comparison to x-ray.

The average standard deviation in MCP images from 2.5 to 2.9 mm is close to the 2.0 for LCR and corresponds to typical standard deviations from distance measurements known from Heil et al. in the range of 1.83–9.1 mm [10].

The precision for selecting central incisor landmarks (*-1apex*, *-1li*, *+1apex* and *+1ls*) was very high in LCR (0.4±0.2 for *+1ls*). This demonstrates a very accurate selection and high level of agreement between the 40 assessors. Although the distance values for the -1apex and -1li in MCPs was in a very good range (1.0–2.2) ±(0.6–1.5), in comparison to the very precise statistics in LCR the t-test was not seen as equivalent.

The authors decided to not test with predefined margins of clinical acceptable ± 2mm [10, 18], since a general predefined margin of acceptance would not show individual precision difference for particular landmarks, as aimed by this study. Despite for point *Ar* a mean deviation of ± 2mm was also confirmed by this study.

The additional spread of approximately 1 mm in mean values in MCP images in comparison to LCR can be observed on average. This difference may be described either by the inaccuracy that was introduced using the MRI modality or due to the missing training in the interpretation of MRI images. Certainly further investigation after proper training of the assessors in the modality is reasonable in next studies.

### Angular measures

The overall average difference between MCP and the gold-standard LCR for angular measurements was within 1.2°, which was within the standard deviation of angular measurements in LCR itself (1.8°).

Since some of the landmarks are essentially tangential construction points, the level of agreement between the assessors in the landmark identification may be smaller in comparison to angular evaluation. This was observable for landmarks *ANS* and *PNS*, which have shown high relative distance values in the metric measurements but lead to small differences in the angular analysis of *S-N/ANS-PNS*.

The gonial angle showed the largest average difference between MCP and LCR of -4.4±7.1°. The interincisal angle showed also unexpected high differences. Both angles didn’t showed statistical similarity in all MCPs. This correlates well to the larger uncertainties of relevant landmarks such as *Ar* or -1apex, which also didn’t show statistical similarity for all MCPs (**Table 4**).

On Average mean deviation between MCPs and LCR in terms of angular measurements was proportionally decreased with longer scan duration (from 1.4° for i = 1 to 0.9° for i = 7). This small improvement may be related to the higher resolution. Eley et al. [9] showed that the overall difference between „black bone”MRIs compared to the gold standard was 1.2°-2.1°, which compares nicely to the 1.2° mean difference that was found in this study. The mean angular difference of 0.54° reported by Heil et al. [11] was not achieved. In contrast to our study they have considered 2 experts in MRI as assessors.

Both mentioned studies reported higher differences in angular measurements for the interincisal angle, which is in line with the findings in our study. The most obvious explanation could be that the landmarks defining the inter-incisal angle lie comparably close to each other and thus the impact of errors on angular changes is exponential.

The LCR is a perspective projection technique which relies fundamentally on the source-detector-distance and the individual position, orientation and size of the specimen within the x-ray system. Thus, registration of 3D MRI or MCP with LCR is a 2D/3D registration problem [20], which was the reason for relative metric evaluations. Even through a typically large source to detector distance of >1.5 m in x-ray systems still two points, which would lie on the same pixel in an orthogonal projection may be projected with several mm distance into the LCR image plane. Therefore, the authors suggest further investigations based on simulations or phantom measurements to evaluate systematic differences in the comparison of the two modalities.

### Strengths and limitations

The speed of our acquisition protocol is striking higher in comparison to other techniques. This has several reasons: first, no time-intensive 3D acquisition is necessary, since the projection from 2D into 3D is directly coded in the slice excitation pulse. Second, the UTE modality with small flip-angles is typically faster since the repetition time is usually also faster. Third, no post-processing e.g. cropping is necessary.

The identification of point *Ar* showed the highest level of uncertainty in this study. In fact the overlay of strong muscles with high signal in MRI reduces the contrast in this area significantly. This impacted also the accuracy of the gonial angle with the highest deviations between MCPs and LCR. One way to increase the contrast in this area would be to use a non-standard excitation pulse, which may not excite areas out of interest for orthodontic treatment. Another option will be to use smaller slice thicknesses also oriented on the left or right side of the midsagittal plane to analyze also facial asymmetries, which can be also an opportunity in comparison to classical x-ray projections. A strength of this study was that a large pool of 40 individual expert assessors was employed in comparison to other studies in this area [9, 10]. By this, it was possible to simulate real-world conditions for landmark identification as well as investigate, by which extent MRI imaging can already be applied for CA without additional training of expert orthodontics. The number of subjects was limited in this technical evaluation. Also the effect of training in MRI/MCP modality was not considered. These aspects should be addressed by further investigations.

## Conclusion

MCP was introduced as a novel protocol based on UTE MRI for cephalometric imaging. The MCP scans have been adjusted in resolution and sampling such that scan times in the range of 5–154 seconds are realized, which were considered as fast in comparison to other known methods [8–10].

Results from the CA were compared on a statistical basis (40 assesors) in comparison to LCR. Hereby images with higher resolution and contrast (e.g. i = 7) showed mean angular deviations up to 0.9°. Lower resolution images with 5–16 seconds (i = 1,2) performed with mean angular deviation up to 1.6° in comparison to LCR.

The aim of this report was to show the potential of this method for further analysis and to demonstrate first technical feasibility of the approach. Benefits of the method are shorter scan times, which may reduce motion artifacts and one shot projection, which provides anatomical information from paramedian planes without post-processing. Further research is needed to analyze the performance of the method for orthodontic questions on a broader basis.

## Supporting information

S1 File Landmark identifikation process. (DOCX)

S2 File Landmark data. (XLSX)

S1 Fig Visualization of landmarks. (PNG)

S2 Fig Visualization of landmarks. (PNG)

S3 Fig Visualization of landmarks. (PNG)

S4 Fig Visualization of landmarks. (PNG)

S5 Fig Visualization of landmarks. (PNG)

S6 Fig Visualization of landmarks. (PNG)

S7 Fig Visualization of landmarks. (PNG)

S8 Fig Visualization of landmarks. (PNG)
