# Supplementary material for: Ultra short time to Echo (UTE) MRI for cephalometric analysis–Potential of an x-ray free fast cephalometric projection technique
Source: PLoS One. 2021 Sep 13;16(9):e0257224. doi: 10.1371/journal.pone.0257224 (PMC8437275; doi:10.1371/journal.pone.0257224)
Supplement: S1 File — (DOCX) [file pone.0257224.s001.docx]

# Data flow

| 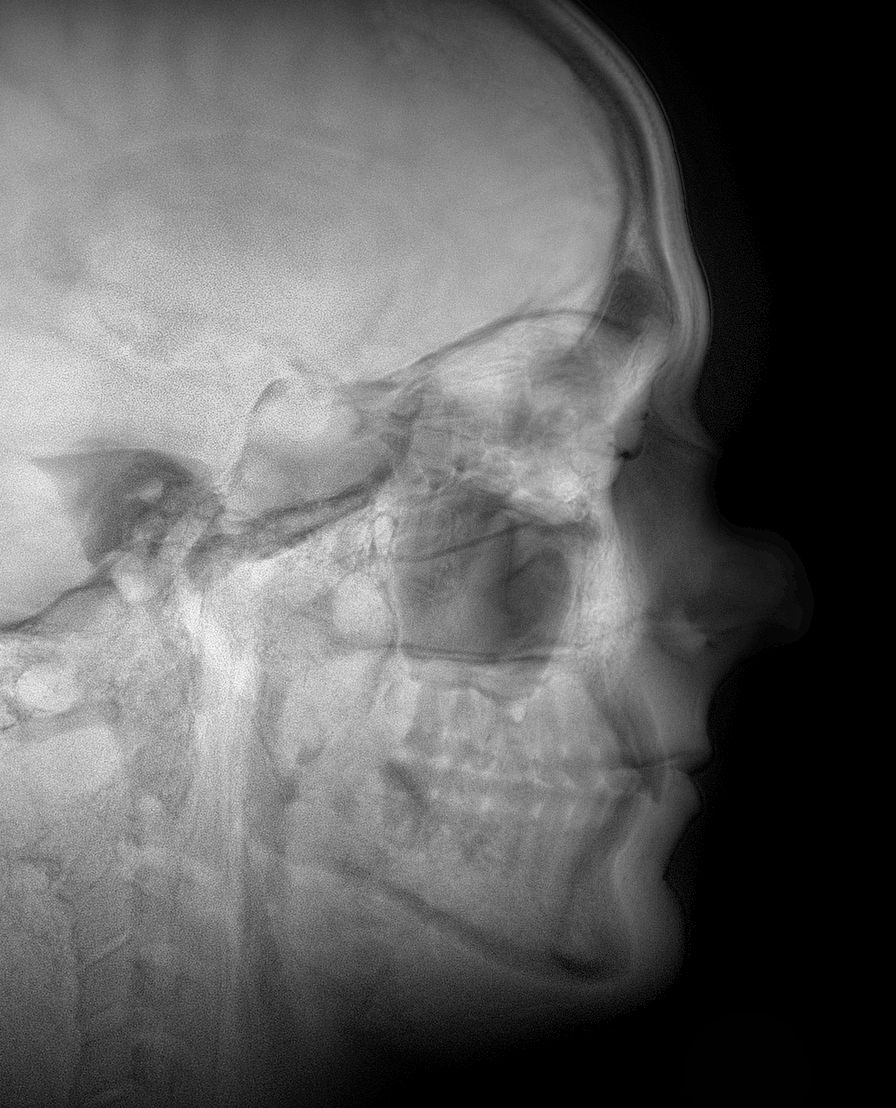 | Image i=1.7 was presented to the assessors in random order. Image i=8 presented last. |
| --- | --- |
| 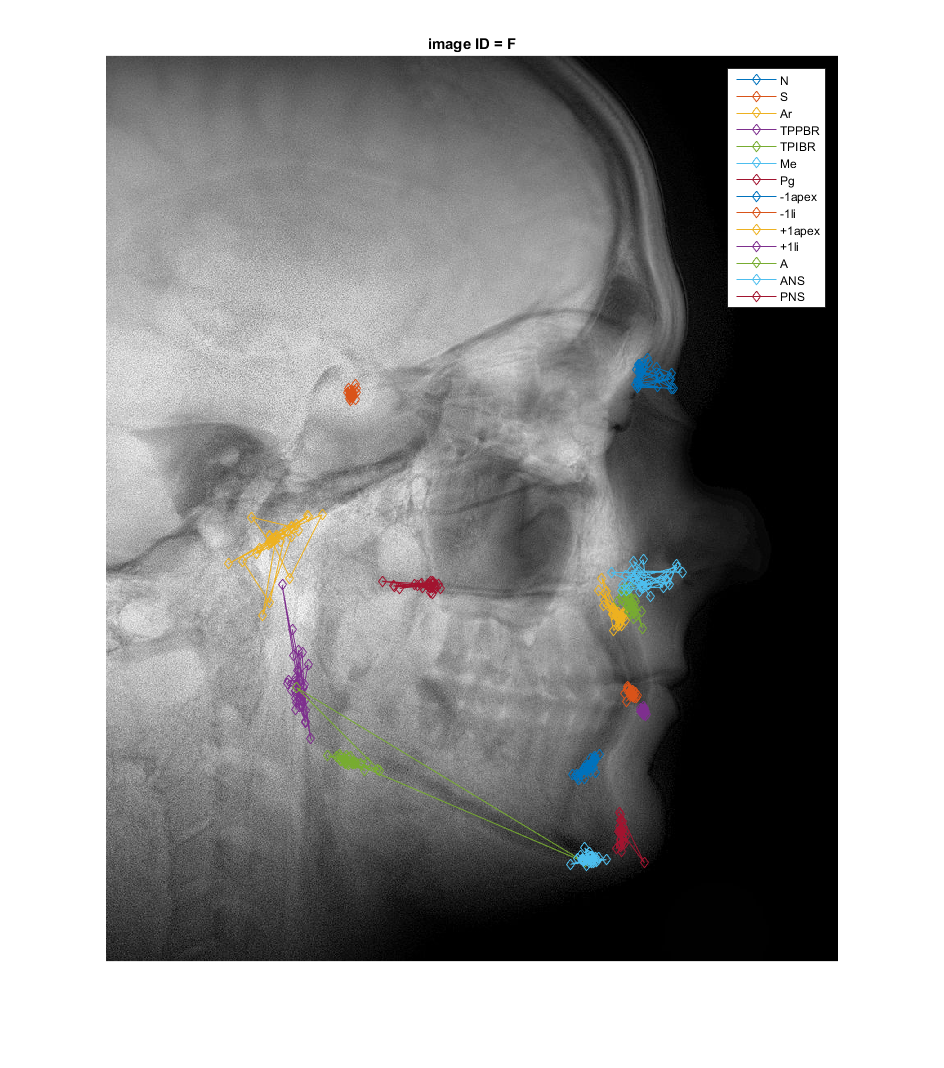 | Assessor selects the individual landmarks.  Points were recorded for all landmarks and lead to a point cloud for each landmark (here Nasion “N”). |
| 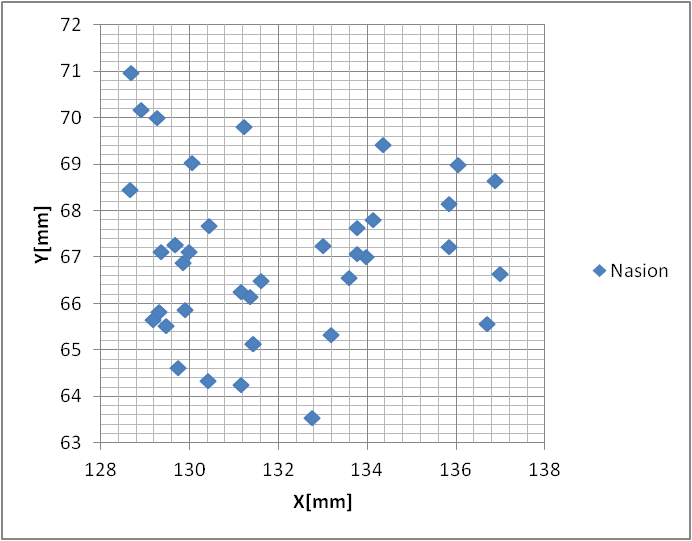 | Distance and angular values are calculated on the basis of the point clouds. |
| 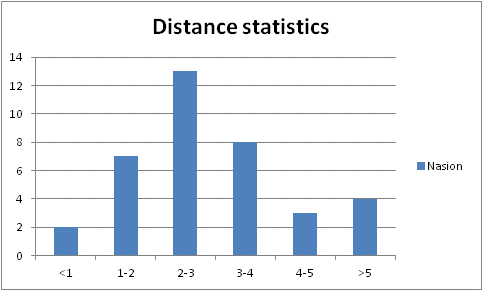 | Statistics are evaluated including check of normal distribution and t-test calculations. |
|  | Angular evaluation is performed and statistical equivalence to x-ray is calculated. |
