# Supplementary figures and images for: Ultra short time to Echo (UTE) MRI for cephalometric analysis–Potential of an x-ray free fast cephalometric projection technique
Source: PLoS One. 2021 Sep 13;16(9):e0257224. doi: 10.1371/journal.pone.0257224 (PMC8437275; doi:10.1371/journal.pone.0257224)

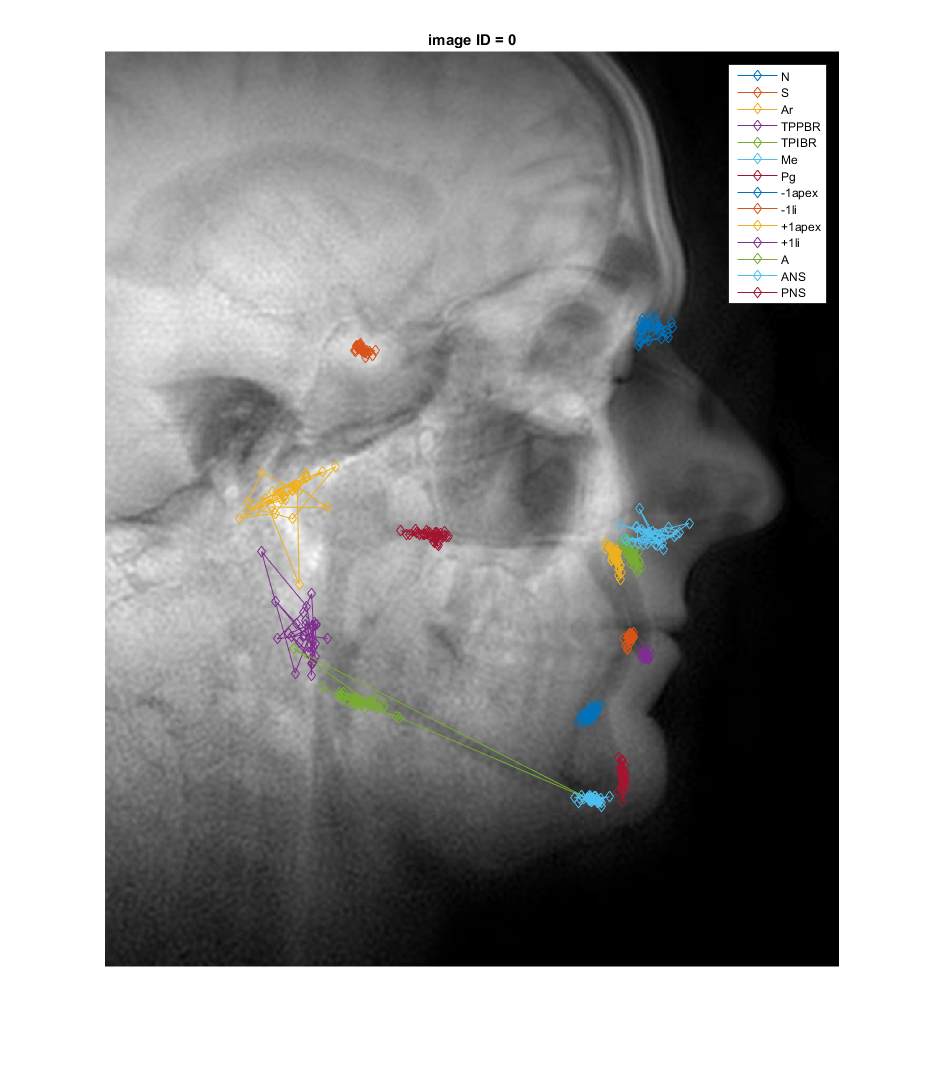

Supplement: S1 Fig — (PNG) [file pone.0257224.s003.png]

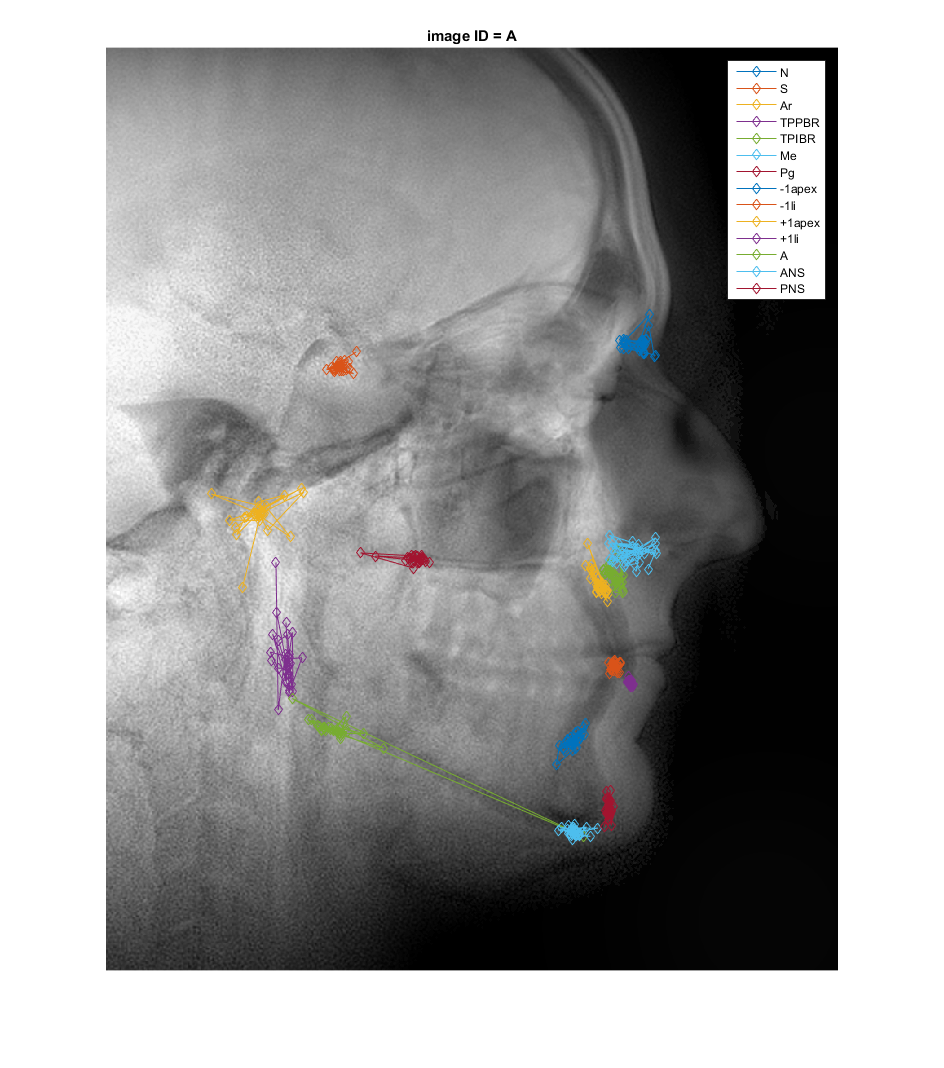

Supplement: S2 Fig — (PNG) [file pone.0257224.s004.png]

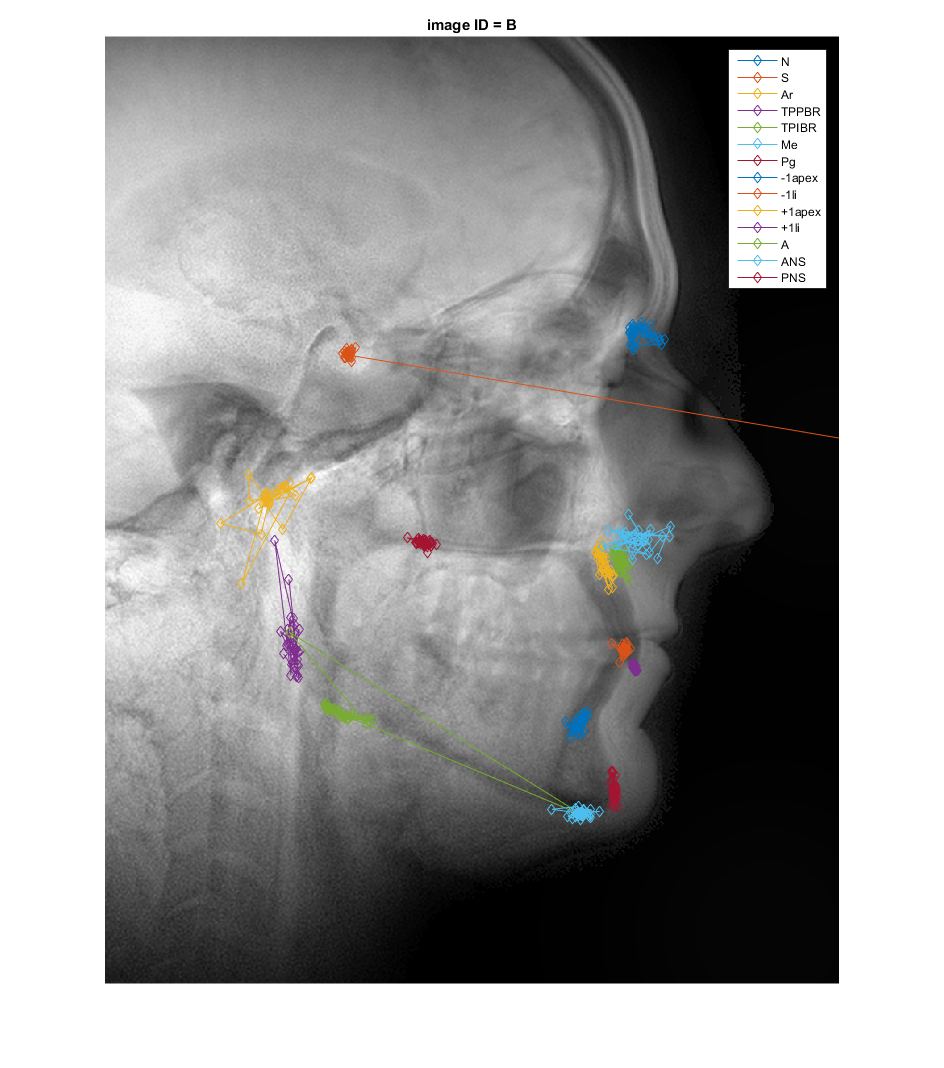

Supplement: S3 Fig — (PNG) [file pone.0257224.s005.png]

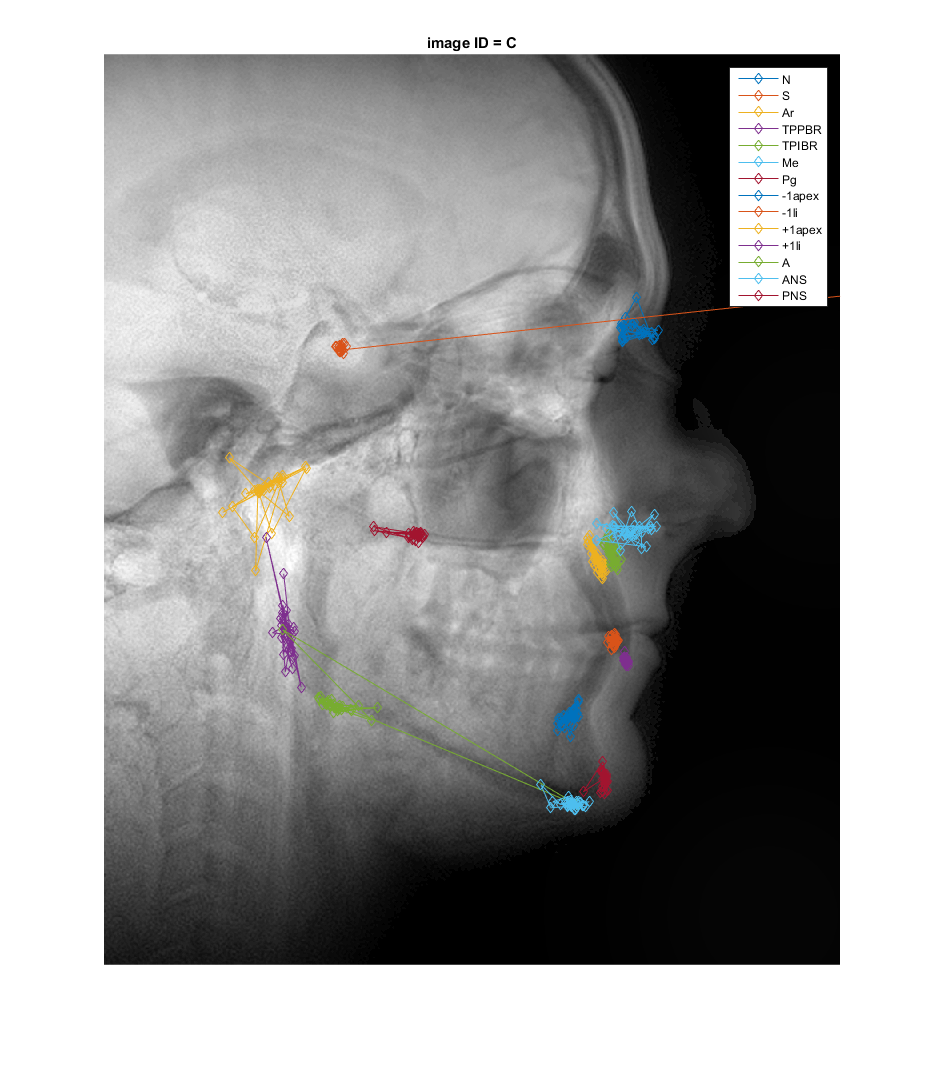

Supplement: S4 Fig — (PNG) [file pone.0257224.s006.png]

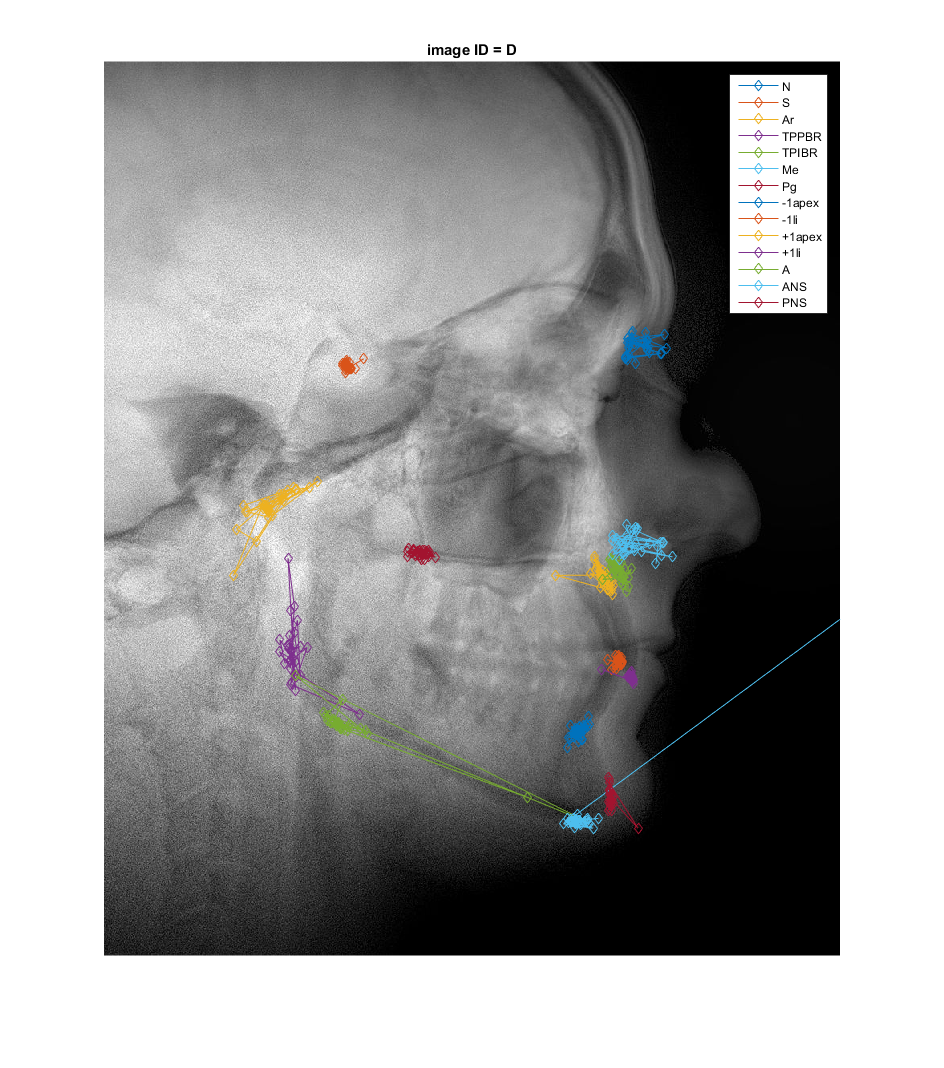

Supplement: S5 Fig — (PNG) [file pone.0257224.s007.png]

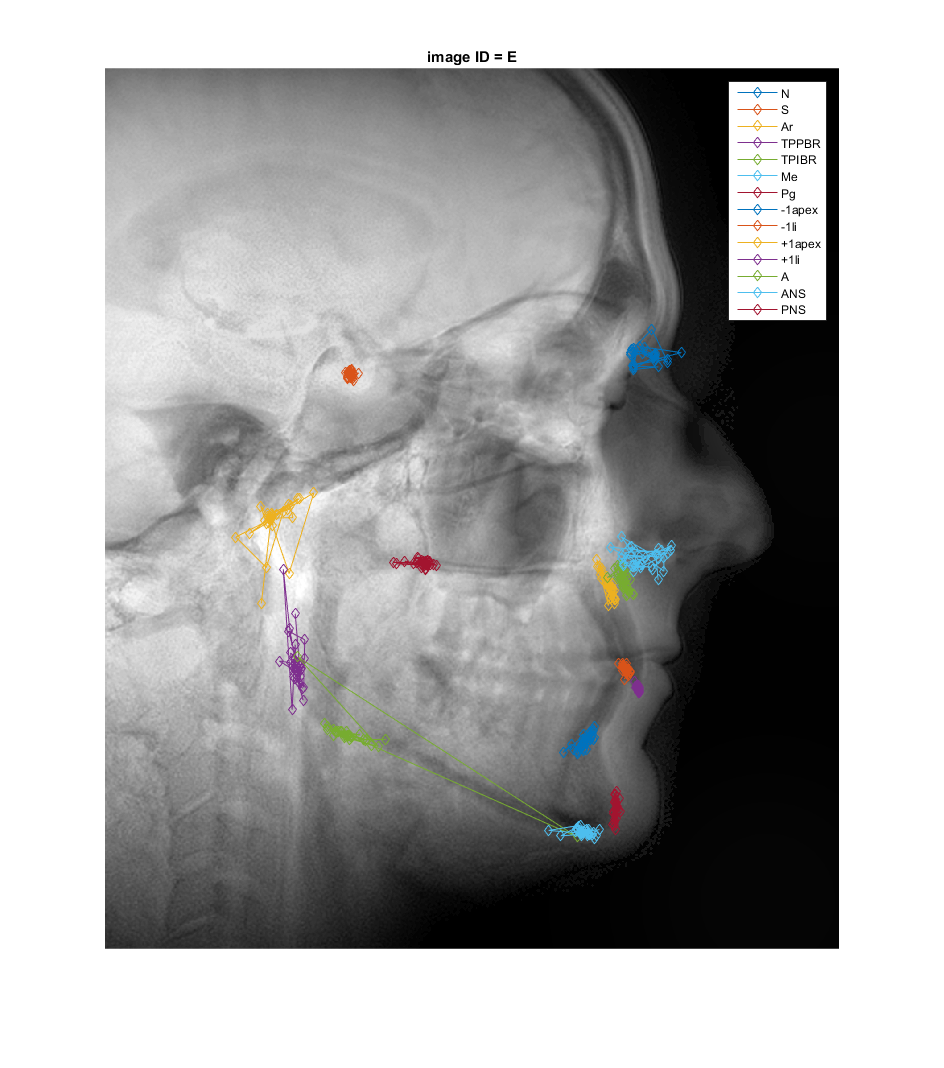

Supplement: S6 Fig — (PNG) [file pone.0257224.s008.png]

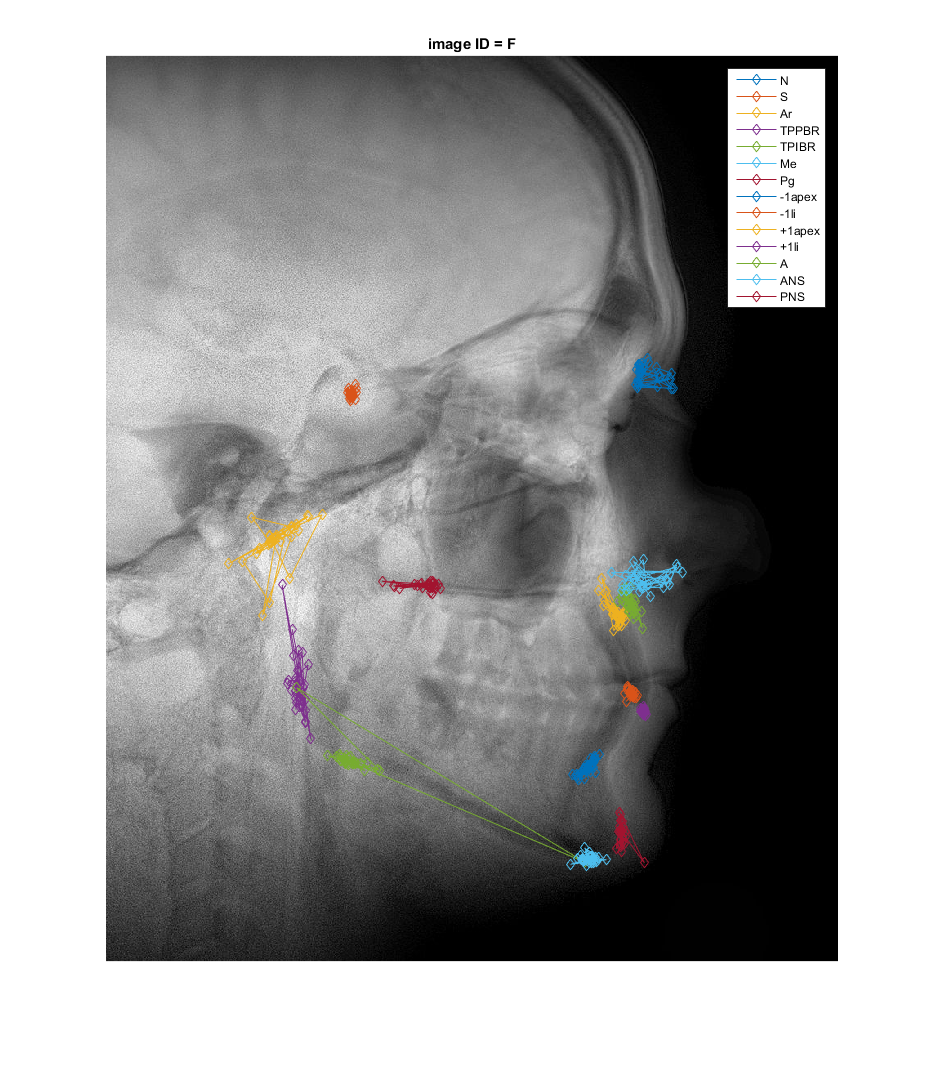

Supplement: S7 Fig — (PNG) [file pone.0257224.s009.png]

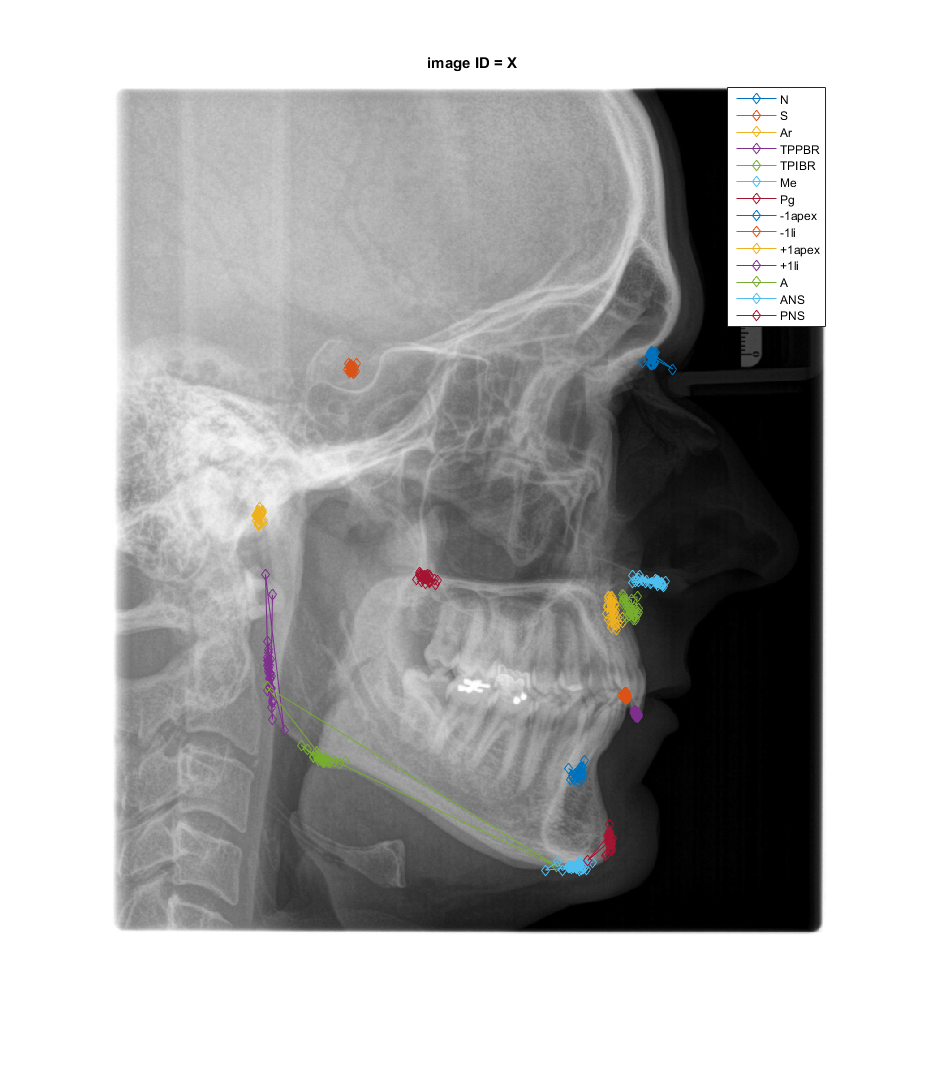

Supplement: S8 Fig — (PNG) [file pone.0257224.s010.png]
